# Asymptomatic and Recurrent Hyperparathyroidism

**Published:** 1989-08

**Authors:** J R Farndon

**Affiliations:** Professor of Surgery, University of Bristol


### Asymptomatic and Recurrent Hyperparathyroidism

**J R Farndon**
Professor of Surgery, University of Bristol

Based on a Presentation to the South West Surgeons Meeting
Barnstaple, 14 October 1988

This article discusses two of the more difficult or controversial aspects of parathyroid disease and the two topics are more strongly related than might at first be apparent. Surgical intervention in patients with subtle biochemical abnormalities and mild symptoms is associated with a higher incidence of failed exploration with persistent disease.

### Symptomatic Disease

*Bone disease* in its florid form, osteitis fibrosa cystica generalizata, is now rarely seen. In 1947 Norris (1) found an incidence of this condition of 91% in those with a parathyroid adenoma. The earliest changes can be detected by X-rays of the hands where subperiosteal erosions can be detected in the phalanges (especially radial aspect of the middle phalanges) and terminal tufts. The skull demonstrates a mottled appearance with lucent cystic areas ('pepper pot skull' in its most florid form) and any bone may demonstrate cystic lesions due to osteoclastomas or brown tumours. These lesions produce skeletal pain and may lead to pathological fractures. Subtle bone changes are rarely detected on skeletal survey and there is little justification for radiology to aid diagnosis unless there are specific symptoms.

*Renal disease* usually manifests as stone production or nephrocalcinosis and the patient may complain of polyuria, polydypsia, ureteric colic, renal pain, haematuria and symptoms of renal tract infections. In a Swedish study in 1986 (2) renal calculi were the presenting marker in approximately 65% of males but in only about 25% of females. The degree of hypercalcaemia was often not severe and a high proportion of patients had chief cell hyperplasia. Successful surgery is more difficult to achieve in this setting and some postulate that this early presentation with mild biochemical abnormality and hyperplastic glands might be the precursor of more severe adenomatous disease (2, 3).

*Hypertension* may be found in many patients with hyperparathyroidism but there are no ready explanations for this. Salahudeen *et al* (4) could find only subtle differences in renal function tests in those with hypertension compared with those without.

*Gastrointestinal symptoms* may relate to peptic ulcer disease, constipation, pancreatitis or gall stones, all of which are variously linked with hyperparathyroidism with varying degrees of certainty. These other conditions all occur commonly and their link may be by pure chance alone although in some the mechanism is clear e.g. peptic ulcer due to hypergastrinaemia from a pancreatic tumour associated with multiple parathyroid adenomas as part of the multiple endocrine neoplasia type I syndrome.

Vague *psychiatric and neuromuscular symptoms* might account for 30% of males and females presenting with hyperparathyroidism (2). These modes of presentation might be dismissed as coincidental. The advent of multichannel blood autoanalysers coupled with a low threshold on the physician's part to initiate investigation if a patient presents in this way results in these people being uncovered or discovered! Joborn *et al* (5), however, have demonstrated that; symptoms can be reversed after parathyroidectomy, their severity is not related to the degree of hypercalcaemia and changes in the turnover of central nervous system monoamines may relate to the symptomatology.

### Asymptomatic disease and the incidence of hyperparathyroidism

It is difficult to be precise on the incidence since this is likely to vary with the population studied and the means of diagnosis. The advent of multichannel biochemical analysers has influenced this observation and allowed the concept of asymptomatic or minimally symptomatic disease. A patient presents to his primary care physician with a specific complaint (not normally associated with hyperparathyroidism) or with non-specific compaints, e.g. malaise, depression, tiredness, and a "blood test" will be drawn in the hope that it will cast light on the nature of the complaint. Whether desired or not a serum calcium determination will be reported and this might be the first intimation of biochemical abnormality. The pursuit of this abnormality is likely to demonstrate other features compatible with primary hyperparathyroidism. In most current series it is exactly this form of the disease which presents. It is presumed that awareness and improved diagnostic facilities have detected and treated almost all patients with severe bone disease. The author has recently had a 53 year old female patient referred directly from the primary care physician (general practitioner) with *all* the necessary diagnostic biochemistry and radiology determined from the practitioner's office. The patient was given a date for surgery and a 2 g adenoma excised giving biochemical correction and resolution of mild, non-specific complaints such as tiredness and malaise.

Further examples of this changing pattern in disease is exemplified by three different studies. Haff and his colleagues sensed this changing pattern in 1970 and reported an incidence of 1 in 2,000 (6). Boonstra and Jackson looking at a blood donor panel estimated the incidence at 1 in 1,000 (7) and more recently in a hospital population the incidence was found to be as high as 1 in 680 (8). In the U.K. the incidence is estimated to be about 25 per 100,000 general population and it has been shown that the majority of these patients will be "asymptomatic" (9). In 1961 the incidence of 'asymptomatic' disease was only 15% (10). The definition of asymptomatic can be very difficult and what might be attributable to old age in an eighty year old would not be acceptable to most forty year olds. Symptoms can develop insiduously over months or years and can so easily be ascribed by a pateint to ageing. It is only after restoration of biochemical normality that a patient might be able to say retrospectively that 'things were not quite right'. A short period in hospital associated with nursing and medical care and a cervical exploration might be no mean placebo however!

Coe and Favus were some of the first to ask 'Does mild, asymptomatic hyperparathyroidism require surgery?' (11) They felt that surgery should be undertaken since renal damage will occur in some untreated patients and that intensive medical follow-up proves a burden. Some have shown, however, that in the short term (about 4 years) there is no sign of deterioration in blood pressure or plasma creatinine comparing age-matched unoperated controls with those who have undergone parathyroidectomy (12). Indeed clodronate sodium (dichloromethylene diphosphonate) has been proposed as a form of medical treatment for hyperparathyroidism particularly in those in whom suppression of bone disease is desirable before surgery or in whom surgery is contraindicated (13).

The debate continues currently. Stevenson and Lyn (14) advocate parathyroidectomy for all patients with asymptomatic mild primary hyperparathyroidism because:

(i) subtle physical and psychological changes are only appreciated on restortion of biochemical normality,

(ii) of the risk of developing renal failure in the long term,

(iii) of the risk of bone loss-especially important in elderly females,

(iv) of the possible contribution of hypercalcaemia to confusion in the elderly,

(v) of the risk of hypercalcaemic crisis in the elderly especially if there is intercurrent illness to produce dehydration,

(vi) of the possibly increased mortality from cardiovascular disease which is increased in incidence.

Not all agree with this policy since the reasons given above are associated with degrees of uncertainty. Heath (15) demonstrates that the workload adopting such a policy would not be inconsiderable—perhaps as many as 170 new patients from Birmingham over 70 years of age with asymptomatic disease. He concludes 'I would find it difficulty on the present evidence to ask a fit, asymptomatic, elderly patient to undergo parathyroidectomy'.

An obstructed femoral hernia requires surgical treatment. The situation is not so clear cut for mild or asymptomatic hyperparathyroidism.

**Recurrent and persistent hyperparathyroidism**

Persistent hyperparathyroidism is the commonest cause of postoperative hypercalcaemia and is continued hypercalcaemia in the immidiate postoperative period or occurring within one year of surgery. Recurrent hyperparathyroidism is defined by Muller (16) as recurrent hyprcalcaemia occurring after the following criteria have been met; (a) identification and biopsy proof of all four parathyroids at the initial operation, (b) complete removal of all abnormal tissue, (c) a normocalcaemic phase of one year or longer and (d) abnormal tissue uncovered at re-exploration at a site of a previously normal gland. True recurrent disease might account for 1% of recurrent hypercalcaemia.

If persistent disease is documented the diagnosis must be checked and confirmed. If hyperparathyroidism is present the next steps require careful thought. There would be no virtue in attempting to localize the abnormal tissue if the patients general condition would not withstand re-exploration and its associated morbidity. If the disease were asymptomatic this too might allow a conservative approach especially if it could be shown that kidneys, bones and eyes were not being damaged by the disease.

If the diagnosis is confirmed and a decision is made to offer re-exploration then most surgeons would use localization procedures preoperatively. This would be carried out consulting previous operation notes, operative maps and histology reports as these can often indicate likely sites of abnormality. If, for example, all glands had been identified and biopsied as normal except the left lower parathyroid then radiologists might use techniques which look in this area to detect intrathyroid masses or mediastinal adenomas.

In earlier years arteriography and selective venous catherization with sampling for parathyroid hormone assay were localization procedures which gave reasonable results. The techniques were costly and invasive and newer developments have allowed other procedures to be adopted. Almost 90% of some series will have localization by these less invasive techniques; ultrasonography: accuracy 76%, sensitivity 82% and positive predictive value 81% or mediastinal computed tomography: accuracy 76%, sensitivity 57% and positive predictive value 80% (17). Others agree that localization procedures are not justified before first exploration but thallium/technetium isotope scans followed by CT scans give reasonable results with specificities of 100% and 71% respectively (18) before reexploration.

If ultrasound is confirmed with fine needle aspiration biopsy for cytology then the accuracy of ultrasound can be improved. The suspect lesion is biopsied and cells studied or the aspirate assayed for parathyroid hormone (Karstrup *et al* 1985) (19) directing the surgeon very precisely in the reoperative site.

Peroperative techniques can be applied. A preoperative infusion of methylene blue stains adenomas and hyperplastic glands a deep purple colour at surgery (20) and intraoperative high resolution ultrasound has excellent sensitivity and positive predictive value (21).

Using these techniques almost 90% of patients will achieve resolution of hypercalcaemia at reoperation. Mortality can be non-existent but there is increased morbidity; 6% temporary recurrent nerve neuropraxia, 4% permanent unilateral cord paralysis and 13% rendered hypoparathyroid (17). The disease site is usually in the neck and with these likely problems the surgery should certainly not be undertaken by the occasional surgeon.

It must be emphasized that failure of initial surgery is nearly always due to an inadequate initial neck exploration. This might be a joint error between surgeon and pathologist, for example, failure to recognize familial hyperparathyroidism or multiple endocrine neoplasia syndromes (22). If lower glands are absent at first exploration then reoperatively the lower pole of the thyroid often harbours a parathyroid adenoma. Intraoperative ultrasound might detect such tumours but often incision of the thyroid lobe or lobectomy will be required (23).

### CONCLUSIONS

Biochemical abnormalities compatible with hyperparathyroidism will continue to be uncovered in patients with no or mild symptoms. Only careful longitudinal studies will provide guidelines for the indications of surgical intervention. Early disease is associated with surgical pathology which is more difficult to treat leading to a higher incidence of persistent disease. Re-exploration is associated with increased morbidity.

These points and the relative rarity of the condition are strong arguments in favour of the disease being treated in a single centre where surgical, pathological and radiological expertise can be developed.

### REFERENCES

1. NORRIS, E. H. (1946) *Arch. Path.* **42**, 261.
2. AKERSTRÖM, G. *et al.* (1986) *World J. Surg.* **10** 696.
3. FARNEBO, L. *et al.* (1988) **12**, 534.
4. SALAHUDEEN, L. K. *et al.* (1989) *Clinical Science* **76**, 289.
5. JOBORN, C. *et al.* (1988) *World J. Surg.* **12**, 476.
6. HAFF, R. C., BLACK, W. C. and BALLINGER, W. F. (1970) *Ann. Surg.* **171**, 85.
7. BOONSTRA, C. E. and JACKSON, C. E. (1971) *Am. J. Clin. Pathol.* **55**, 523.
8. HARROP, J. S., BAILEY, J. E. and WOODHEAD, J. S. (1982) *J. Clin. Pathol.* **35** 395.
9. MUNDY, G. R., COVE, D. H., FISKEN, R. *et al.* (1980) *Lancet.* **1**, 1317.
10. KEYNES, M. (1961) *Br. Med. J.* **i**, 239.
11. COE, F. L. and FAVUS, M. J. (1980) *N. Engl. J. Med.* **302**, 224.
12. Van't Hoff, W., BALLARDIE, F. W. and BICKNELL, E. J. (1983) *Br. Med. J.* **287**, 1605.

13. DOUGLAS, D. L., KAMIS, J. A., PATERSON, A. D. *et al.* (1983) *Br. Med. J.* **286**, 587.
14. STEVENSON, J. C. and LYNN, J. A. (1988) *Br. Med. J.* **296**, 1017.
15. HEATH, D. (1988) *Br. Med. J.* **296**, 1398.
16. MULLER, H. (1975) *Br. J. Surg.* **62**, 556.
17 GRANT, C. S. *et al.* (1986) *World J. Surg.* **10**, 555.
18. KRUBSACK, A. J. *et al. World. J. Surg.* **10**, 579.
19. KARSTRUP S. *et al.* (1985) *Br. Med. J.* **290**, 284.
20. BAMBACH, C. P. and REEVE, T. S. (1978) *Aust. NZ. J Surg.* **48**, 314.
21 KERN, K. A. *et al.* (1987) *World J. Surg.* **11**, 579.
22 CLARKE, O. H. *et al.* (1976) *Ann. Surg.* **184**, 391.
23 WHEELER, M. H. *et al.* (1987) *World J. Surg.* **11**, 110.

